# Factors for Supporting Primary Care Physician Engagement With Patient Apps for Type 2 Diabetes Self-Management That Link to Primary Care: Interview Study

**DOI:** 10.2196/11885

**Published:** 2019-01-16

**Authors:** Julie Ayre, Carissa Bonner, Sian Bramwell, Sharon McClelland, Rajini Jayaballa, Glen Maberly, Kirsten McCaffery

**Affiliations:** 1 Sydney Health Literacy Lab Sydney School of Public Health, Faculty of Medicine and Health The University of Sydney Sydney Australia; 2 Ask, Share, Know: Rapid Evidence for General Practice Decisions Centre for Research Excellence The University of Sydney Sydney Australia; 3 Western Sydney Diabetes Western Sydney Local Health District Blacktown Australia; 4 School of Medicine Western Sydney University Blacktown Australia; 5 Sydney School of Public Health Faculty of Medicine and Health The University of Sydney Sydney Australia

**Keywords:** diabetes mellitus, type 2, electronic health records, telemedicine, mobile apps, general practitioners, physicians, primary care, self-management, qualitative research, translational medical research

## Abstract

**Background:**

The health burden of type 2 diabetes can be mitigated by engaging patients in two key aspects of diabetes care: self-management and regular contact with health professionals. There is a clear benefit to integrating these aspects of care into a single clinical tool, and as mobile phone ownership increases, apps become a more feasible platform. However, the effectiveness of online health interventions is contingent on uptake by health care providers, which is typically low. There has been little research that focuses specifically on barriers and facilitators to health care provider uptake for interventions that link self-management apps to the user’s primary care physician (PCP).

**Objective:**

This study aimed to explore PCP perspectives on proposed features for a self-management app for patients with diabetes that would link to primary care services.

**Methods:**

Researchers conducted 25 semistructured interviews. The interviewer discussed potential features that would link in with the patient’s primary care services. Interviews were audio-recorded, transcribed, and coded. Framework analysis and the Consolidated Criteria for Reporting Qualitative Research checklist were employed to ensure rigor.

**Results:**

Our analysis indicated that PCP attitudes toward proposed features for an app were underpinned by perceived roles of (1) diabetes self-management, (2) face-to-face care, and (3) the anticipated burden of new technologies on their practice. Theme 1 explored PCP perceptions about how an app could foster patient independence for self-management behaviors but could also increase responsibility and liability for the PCP. Theme 2 identified beliefs underpinning a commonly expressed preference for face-to-face care. PCPs perceived information was more motivating, better understood, and presented with greater empathy when delivered face to face rather than online. Theme 3 described how most PCPs anticipated an initial increase in workload while they learned to use a new clinical tool. Some PCPs accepted this burden on the basis that the change was inevitable as health care became more integrated. Others reported potential benefits were outweighed by effort to implement an app. This study also identified how app features can be positively framed, highlighting potential benefits for PCPs to maximize PCP engagement, buy-in, and uptake. For example, PCPs were more positive when they perceived that an app could facilitate communication and motivation between consultations, focus on building capacity for patient independence, and reinforce rather than replace in-person care. They were also more positive about app features that were automated, integrated with existing software, flexible for different patients, and included secondary benefits such as improved documentation.

**Conclusions:**

This study provided insight into PCP perspectives on a diabetes app integrated with primary care services. This was observed as more than a technological change; PCPs were concerned about changes in workload, their role in self-management, and the nature of consultations. Our research highlighted potential facilitators and barriers to engaging PCPs in the implementation process.

## Introduction

For people with diabetes, self-management (including medication adherence, physical activity, healthy diet, and weight management) is a key aspect of care that can mitigate long-term complications of diabetes [1-5]. Because diabetes is a progressive condition, regular interactions with health professionals are important for medical feedback on self-management (such as glycated hemoglobin levels), education, adaptation of the care plan (including adjustment of medications as the condition progresses), and monitoring and treatment of long-term complications [6,7]. As such, self-management and care provided by health professionals are interrelated, and this should ideally also be reflected in clinical diabetes interventions, for example, by fostering ongoing communication between the health care provider and patient to facilitate their respective roles in diabetes care.

As mobile phone ownership increases [8,9], a self-management app that can also be used during consultations could achieve this goal. Mobile phone apps are already available to help people with diabetes engage in self-management [10], and many collect patient data that is highly relevant to the health care provider, including self-monitoring data for blood glucose, physical activity, and diet [11]. Health care providers value self-management apps because they perceive that they encourage patient engagement, provide them with a deeper and more reliable understanding of their patients’ behaviors, and improve communication during consultations by providing visualizations of patient data [12-15].

However, despite the potential benefits of online health technologies (including apps), implementation on a large scale remains a key challenge. Research suggests that their effectiveness is often limited by poor uptake and sustained use by health care providers [16,17]. Recent systematic reviews have suggested that key barriers for health care providers are increased workload and disruption to existing clinical processes and staff roles as well as concerns about remuneration, data security, and liability [16,18-20].

Some barriers may specifically relate to self-management apps. This could include the overwhelming complexity of the data that is available to health care providers, provider responsibility to respond to shared self-monitoring data, and health care provider perceptions of poor motivation on the part of patients [12,14]. Overcoming the challenge of poor provider uptake is crucial as strong provider endorsement is in turn a key factor for patient uptake of online tools [21].

Our study aimed to build on these findings by investigating primary care physician (PCP) perspectives on proposed features for a self-management app for people with type 2 diabetes that is linked to their PCP’s care plan. This will provide a more specific understanding of how PCPs conceptualize their role in providing care to their patients who have type 2 diabetes and how this role could be better supported by an app.

## Methods

### Participants

PCPs were recruited from a pool of 50 clinics in Sydney, Australia, that had elected to engage in joint specialist case conferencing, an initiative implemented through the Western Sydney Primary Health Network in an area with culturally and linguistically diverse patient populations. During case conferencing, the PCP discusses diabetes management with the patient in conjunction with an endocrinologist and a credentialed diabetes educator. PCPs were invited to participate in the interview with a view to informing the design of an app developed by a group of collaborating local health authorities called Western Sydney Diabetes. Purposive sampling ensured a diverse range of gender, years of experience, and cultural backgrounds to reflect the broader PCP population in Western Sydney (see Table 1).

### Procedure

After providing written consent to participate, JA conducted semistructured interviews for approximately 25 minutes in each PCP’s consultation room. Interviews were conducted between November 2017 and June 2018. Questions were based on an interview schedule that was piloted with PCPs prior to this study (Multimedia Appendix 1). Questions pertained to how the PCP currently helps patients to self-manage diabetes and their attitude toward diabetes apps. Participants were also asked for feedback on several specific app features:

- Transfer a patient’s individualized care plan into the app
- Export self-monitoring data to PCP software
- Prompt patient to see their PCP (for example, if there is a pattern of high blood glucose readings)
- Send reminders to book cycle of care appointments (for example, PCP check-ups and eye and foot checks)
- Contain bundles of educational material including videos that can be sent to the patient
- Produce a summary report of blood glucose self-monitoring to be used by the PCP during the consultation (see Multimedia Appendix 1)

Interviews were audio-recorded and transcribed verbatim. Ethical approval was obtained from the University of Sydney Human Research Ethics Committees (project number 2017/224) and Western Sydney Local Health District (reference number 5092 AU RED LNR/17/WMEAD/140).

### Analysis

**Figure 1:**
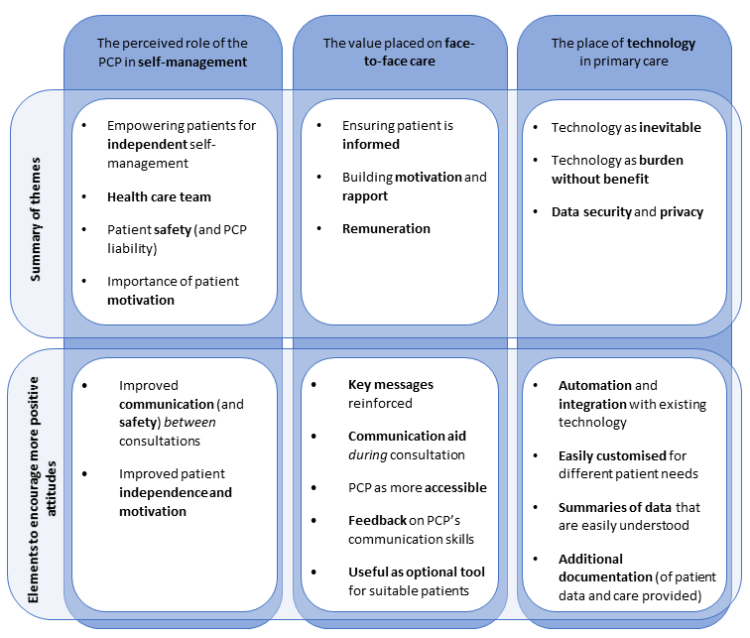
Mind map of themes. PCP: primary care physician.

Interviews were analyzed using framework analysis, a matrix-based approach to thematic analysis [22], which involved 5 steps: familiarization with the data, indexing, collating similar codes into themes, charting data into a thematic framework, and synthesis and interpretation. Rigor was addressed through indexing a subset of data across 2 researchers, a continuous process of comparing concepts and themes to data, and discussion of potential themes across authors. The project team concluded that theoretical saturation was reached after 25 interviews, where variation in PCP perspectives could be adequately explained through 3 overarching themes (Figure 1).

**Table 1:** Participant descriptive characteristics.

Characteristics		Total, n (%)	
**Gender (n=25)**			
	Female		14 (56)
	Male		11 (44)
**Years qualified as a PCP^a^** **(n=24)**			
	<10		5 (20)
	10-19		8 (32)
	≥20		12 (48)
**Country of birth (n=24)**			
	Australia		6 (25)
	Sri Lanka		5 (21)
	India		4 (17)
	Bangladesh		2 (8)
	Philippines		2 (8)
	Other^b^		5 (21)
**Languages spoken (n=24)**			
	English only		4 (17)
	Tamil		5 (21)
	Sinhalese		3 (13)
	Chinese		3 (13)
	Hindi		3 (13)
	Filipino		2 (8)
	Other^c^		8 (33)

^a^PCP: primary care physician.

^b^Other includes countries of birth listed by 1 PCP: South Africa, Afghanistan, Malaysia, Fiji, United Kingdom.

^c^Other includes languages spoken by 1 PCP: Afrikaans, Bangla, Bengali, Dari, Kannada, Malay, Swahili.

## Results

### Overview

Of the PCPs from 12 clinics who were interviewed, 83% (20/24) spoke a language other than English and 80% (20/25) had been qualified as a PCP for at least 10 years (Table 1).

Most PCPs were open to using a mobile phone app for diabetes self-management in their clinic. Each theme below constitutes a set of beliefs that contributed to these attitudes toward proposed features for a self-management app (Figure 1):

- Perceived role of the PCP in self-management of type 2 diabetes
- Value placed on face-to-face care
- Place of technology in primary care

PCPs often shared similar beliefs and values across these 3 themes regardless of their cultural backgrounds. However, there was substantial variation in perceptions of whether an app would support them in their work. Therefore, each theme also highlights the positive mindsets of PCPs who perceived an app would support the care they currently provide.

### Theme 1. Perceived Role of the Primary Care Provider in Self-Management of Type 2 Diabetes

#### Primary Care Physician Perspective

PCPs emphasized that ongoing self-management was a key aspect of care for their patients with diabetes. However, many believed that this was outside their control and that ultimately the patient must take responsibility for self-management.

Well I think it’s very important to think medical people want to do what they can, but it’s really what people do 24 hours of the day, 7 days a week.

PCP07, male, speaks a language other than English (LOTE), practicing ≥20 years

Instead, PCPs perceived that their role was to increase patient capacity for safe and independent self-management. They built this capacity by providing medical advice, general self-management education, and specific feedback (for example, on patterns of blood glucose levels). Several PCPs saw more detailed self-management education as the role of practice nurses, dieticians, or diabetes educators.

App features that expanded PCP responsibilities related to patient self-management raised concerns about liability. For example, many PCPs did not endorse an app feature that would notify them in real time about patients’ blood glucose readings. PCPs perceived that this feature might risk patient safety, particularly if it gave patients the impression that their doctor was actively monitoring their readings. As a result, PCPs anticipated that this would create moral or legal obligations to respond in a timely manner.

...probably I don’t want to receive the data on a regular basis. I think there is a chance that the clinician might miss it. Or if they’ve gone on a holiday or if they haven’t checked the data or anything. So there’s a risk that they could be missed.

PCP13, male, speaks a LOTE, practicing 10 to 19 years

Crucially, some PCPs also perceived that a key limitation of an app was that it did not overcome the initial challenge of persuading patients to take responsibility for their self-management. They argued that an app would only be useful for patients who were already independent and therefore would not target those who most needed to engage in self-management care.

My problem with these apps...we are already selecting a group of people who are going to be motivated enough to put this data into the app...whereas the majority of patients, or the people we have trouble with...who are not bothered to exercise and things, I’m not sure this is going to...convince them to do it...if they can’t walk for half an hour will they take the trouble of putting all this in the mobile app? That’s where my skepticism is.

PCP17, female, speaks a LOTE, practicing ≥20 years

#### Facilitating Features

Despite these concerns, several PCPs perceived that some app features could improve patient safety and independence. For example, PCPs were more positive about an app when they saw it as an opportunity to improve patient safety between consultations. This was particularly important for patients they only saw intermittently.

I think we can deal with the problem before it becomes out of hand and then we can fix the problem on the patient’s point of view or modify the medications if needed to, and so it’s a win, win situation for both.

PCP19, male, speaks a LOTE, practicing ≥20 years

PCPs also preferred features that placed the onus on the patient to take action. This included notifications to patients about patterns in blood glucose readings (where notifications were generated automatically by algorithms) and automated reminders for check-ups.

Last, PCPs who were more positive about using an app perceived that most of their patients engaged in self-management, at least to some degree. As such, they anticipated that an app could benefit patients by increasing motivation between consultations and capitalizing on transient moments of greater motivation.

...So when they’ve got a little bit of motivation in one perspective, or like for a particular problem, you want to try and foster that as soon as it comes on board.

PCP04, male, speaks a LOTE, practicing <10 years

### Theme 2. Value Placed on Face-to-Face Care

#### Primary Care Physician Perspective

All PCPs greatly preferred face-to-face care to online care. Many perceived that face-to-face care enabled them to make sure patients actually took in the information from educational materials. They perceived that they could tailor the information for the patient more easily in person (for example, by presenting information in small amounts, emphasizing the most important points, and checking understanding). PCPs also believed it was a more effective platform to ensure that the patient was at the very least exposed to appropriate information, whereas links could easily be ignored or forgotten.

Because you can send patients the link online...you can give them a lot of information. Don’t know how much of it they’ve actually looked at. That’s probably the biggest limitation, is not knowing exactly what they’ve looked at overall.

PCP15, male, other demographic data missing

Some PCPs also expressed concern that an app would undermine the patient-physician relationship. They argued that face-to-face meetings were important for developing rapport with the patient. One PCP explained how he personalized care with his patients by discussing their motivation to engage in their health.

...so I think it’s important to ask the patient, what matters to you? Well, I don’t want to end up like my Mum...Or I don’t want to go blind...Or I don’t want my kidneys to fail. So...ok, so how is it day-to-day what we can take steps to manage that? So you’ve got to find out what motivates the patient.

PCP02, male, speaks only English, practicing <10 years

A small number of PCPs (n=4) also identified that there was a risk that they may not be paid for time spent delivering care online because there was no existing process.

...we give good care but we also like to be acknowledged for that care and remunerated, appropriately.

PCP01, female, speaks a LOTE, practicing ≥20 years

#### Facilitating Features

Conversely, PCPs were more positive when they perceived that an app would be a welcome adjunct to face-to-face care that could address existing challenges. For example, PCPs were interested in brief educational materials that would reinforce the key messages discussed during the consultation. This was particularly important when patients found it difficult to take in information during the consultation because they were distressed or overwhelmed.

...I realize you can tell people something and it just goes straight over their head. Or you’ve given them some bad news and then you tell them something else, it just hasn’t registered...I guess in one sense, anything that helps information being given to the patient is good...

PCP03, male, speaks only English, practicing ≥20 years

Others identified that an app could help overcome difficulties conveying information during the consultation itself. PCPs discussed how resources that use pictures or that are available in the patient’s first language and simple graphs of blood glucose levels could help to convey messages during the consultation and open up discussions about barriers to lifestyle change and the complications of diabetes.

So I think things that are visual are good ‘cause patients, they don’t like all these numbers... So things like this [pie chart] is helpful. So if I can say, all right so the green bit is you in the normal range, but the blue bit is when your readings are too high...we need to try and get your green bits to be a bigger portion...

PCP18, female, speaks a LOTE, practicing <10 years

In contrast to the perception that an app would weaken rapport, some PCPs argued that an app that is linked to the patient’s health care provider could foster a stronger connection with the patient. They discussed how it would create a sense of accessibility to the PCP and encourage patients with patterns of high or low blood glucose readings to see their doctor more frequently.

...I’ve got a lot of patients who I have a lot of trouble... getting in with their numbers. And I think those patients would benefit from something like this...So, yeah, if it’s something they’re engaging in and they’re getting those numbers and they see it and it prompts them to come in and discuss it with me, I think that’d be a good thing.

PCP16, female, speaks a LOTE, practicing <10 years

A few PCPs also saw an app as a tool to improve their communication skills. These PCPs anticipated that some patients may respond to notifications about high or low blood glucose levels by inundating the clinic with inquiries. They perceived that this could be a reflection of poor communication with the patient, particularly in terms of setting clear expectations, providing information and checking understanding.

...if they have more questions it’s probably because they’ve not been given the correct information in the first place. So it’s almost an aid to [PCP] doing their job properly....is how I view it.

PCP14, male, speaks a LOTE, practicing 10 to 19 years

Last, PCPs were more positive when they perceived an app as an optional tool which patients could elect to use. Many PCPs perceived an app could be very useful for patients who were younger or more familiar with mobile phones but would be of limited use with others, primarily older patients who did not regularly use apps or who had significant vision problems (a common complication of diabetes).

### Theme 3. Place of Technology in Primary Care

#### Primary Care Physician Perspective

Most PCPs anticipated that an app would increase the burden of clinical care. They believed that there would be increased workload initially while they learned to use an app, as well as ongoing time required to provide care remotely. Interestingly, despite this shared awareness about the burden of a new clinical technology, PCPs varied substantially in their attitudes. Several PCPs accepted the burden, believing that clinical practice was inevitably shifting towards digital health and mobile phone technologies.

I mean that’s a bridge we’re actually going to have to cross anyway. That’s the reality. So, I mean, the way I’d phrase it is, that’s a burden we’re going to have to take on board, given how technology’s going.

PCP04, male, speaks a LOTE, practicing <10 years

Those with negative attitudes perceived that potential benefits would be outweighed by the effort needed to implement an app. Some also perceived that an app would add unnecessary complexity to their work. For many of these PCPs the current appointment reminder systems and blood glucose logbooks were sufficient.

...they’re not that computer savvy, especially the older generations. In which case it’s more confusing for them and for me, because then I won’t know under what circumstances there was a high BSL. Maybe it was another reason...like it’s almost more confusing sometimes.

PCP14, male, speaks a LOTE, practicing 10 to 19 years

...every time there’s a little bit of fatigue when ...[local health district] or, when [health body] comes up with yet another initiative, it’s like well I’ve always been doing it this way, I’ve trained on this way and now my life is about to get even more complex.

PCP02, male, speaks only English, practicing <10 years

Last, only two PCPs raised issues about data security and privacy. They perceived that they would be held personally responsible if they endorsed and used an app that did not adhere to government privacy policies.

#### Facilitating Features

PCPs were more positive when they perceived that burden would be mitigated. For example, PCPs valued app functions that were largely automated and that were integrated with existing technology and practices.

...[my existing online interface that integrates consultation bookings and patient reminders with practice software] is really good because it actually talks to my software, even though it’s a Web app, it’ll actually talk to my software, then a recall reminder has been sent. So then it doesn’t mean I have to go to something, type it in, go to something else, type it in.

PCP02, male, speaks only English, practicing <10 years

It is worth noting that PCPs encouraged complexity to ensure that variability in patient characteristics and goals was accommodated. For example, PCPs reported that care plans were different for patients who were newly diagnosed, those who were transitioning to insulin, and those who switched to a new medication. As such, many PCPs preferred options that allowed them to customize the frequency of reminders and the presentation of patient data. One PCP even highlighted that the colors used for high and low blood glucose readings should reflect whether care was focused on preventing hypoglycemia or preventing long-term complications.

...But I think that’s more practitioner-dependent and how you practice your medicine... If the overall aim of the app is to improve diabetic control then the highs go red. If the overall aim of the app is to prevent complications from diabetes, especially hypoglycemia, then the hypoglycemia end up red.

PCP04, male, speaks a LOTE, practicing <10 years

PCPs also viewed apps more favorably when they perceived additional benefits that an app could provide. For example, PCPs valued features that would analyze patient data and summarize the relevant information so that it could be understood quickly. One PCP discussed that if summary data were available before the consultation, she could make better use of her time with the patient.

I also think if we only get [patient data] in the consult there’s time constraint. We only get 15 to 20 minutes and we won’t necessarily have time to go through all the results and discuss it with the patient and come up with a management plan. So I think if...we can go through [summary data] before we see the patient and we can also have a plan formulated because then we can just discuss it with them and manage it.

PCP22, female, speaks a LOTE, practicing 10 to 19 years

Another perceived secondary benefit of an app was improved documentation for patient data and records of care. PCPs perceived that improving patient data would increase patient accountability. They perceived that improving the documentation of care would form a stronger basis for remuneration and improve care that was shared with other health professionals.

...So if it’s going to be arranged in such a way that the patient uses this app and sends messages to the doctor and the doctor can use that particular opportunity to say that “look this is the care that I’ve given” and use that as an outcome-based visit without seeing the patient face-to-face.

PCP01, female, speaks a LOTE, practicing ≥20 years

## Discussion

### Principal Findings

This study explored PCP perspectives on proposed features for a diabetes self-management app that would be linked to their practice software. Our analysis indicated that these attitudes were underpinned by perceived roles of PCPs in diabetes self-management, the role of face-to-face care, and the anticipated burden of new technologies in their practice. This study also identified how app features can be positively framed, highlighting potential benefits for PCPs in order to maximize PCP engagement, buy-in, and uptake.

The barriers and facilitators identified in this study can be incorporated into an implementation framework. For example, normalization process theory [23] suggests that 4 factors lead to successful uptake and sustained use of new technologies. This study identified several strategies related to coherence (how consumers understand a new technology within the context of existing systems), cognitive participation (how consumers engage with and commit to using a new technology), and collective action (perceived impact of the new technology on workflow, workload, roles, responsibility and training) (Table 2). The fourth factor, reflexive monitoring, relates to technologies that have already been implemented.

### Comparison With Prior Work

The findings in this study identified three key barriers that are specific to uptake of diabetes self-management apps that link to the health care provider. First, there was a clear tension between avoiding an increase in workload and the need for app functions and settings that can be customized to the diverse clinical goals of patients with diabetes. For example, PCPs perceived it was important to have different schedules of prompts when introducing new medication compared to regular blood glucose self-monitoring. A balance between these aspects is needed as workload is a key barrier to provider uptake [16].

Second, PCPs challenged the idea of real-time notifications of patient data. PCPs understood the theoretical value of real-time notifications but perceived that this feature would fail in real clinical settings and could actually put patient safety at risk. This has been identified in previous research on digital self-monitoring [14]. Furthermore, many PCPs voiced that this would not support them in their goal to build patient capacity for independent self-management. Alternatives such as automated prompts directing the patient to see their PCP may be a more realistic option.

Third, some PCPs argued that even if an app is effective, the benefits would be severely limited if it were only suitable for patients who were already motivated, had sufficient familiarity with mobile phone apps, and had adequate vision. However, it should also be noted that mobile phone ownership is high in Australia, with the greatest increases in ownership seen in older age groups [8]. Other research has also reported that health care providers tend to underestimate patient motivation for lifestyle management [24]. Regardless, it will be important for future work to establish how to identify patients who will benefit most from an app and whether other interventions might be more suitable for less motivated patients.

The other barriers identified in this study were less specific to diabetes self-management and are consistently reported in research on implementation of online health interventions. For example, increased workload and changes to scope of practice are common factors for poor uptake of new health technologies [12,13,16-20]. PCPs also suggested that face-to-face care was important for developing rapport with the patient and ensuring that the patient had understood important information. Similar concerns were highlighted in a review of telehealth interventions for patients with heart failure [18]. In addition, face-to-face care is often valued by health professionals because it is perceived as the main method of remuneration [12,24]. However, in our study only four of the PCPs raised this issue.

**Table 2:** Summary of themes and how suggested strategies relate to normalization process theory.

Theme	Description	Examples of suggested strategies by normalization process theory component		
		Coherence	Cognitive participation	Collective action
Theme 1. Perceived role of the PCP^a^ in self-management of type 2 diabetes	PCP goal is to facilitate independent self-management for patients with diabetes Care is shared across practice staff Patients aren’t motivated to self-manage	Explain where the goals of the app overlap or are likely to differ from PCP goals to support patients Explain any medicolegal risks, particularly in terms of remote monitoring of blood glucose Explain how staff can continue existing roles through the app Explain how to identify patients who have enough baseline motivation/independence to use the app	Explain how an app can address existing challenges Explain how the app can bolster patient motivation between consults	Explain that the Intervention will be available to various staff including nurses
Theme 2. Value placed on face-to-face care	Face-to-face care is valuable PCPs are remunerated primarily through face-to-face care Patients don’t use mobile phones	Explain how the app is an optional additional tool; it does not replace face-to-face care. Provide guidance on how to best identify patients who are suited to the app Be explicit about whether/how work conducted through the app will be remunerated	Explain how app can improve efficiency of analysis of self-monitoring data Explain how app can facilitate communication during consultation and promote the take home message Explain how app can prompt patient to visit doctor	Not applicable
Theme 3. Place of technology in primary care	This is just another thing we have to learn to use (with little added benefit) It will take a lot of time to learn to use the app Patients are not one-size-fits-all Data security and privacy	Be explicit about whether/how work conducted through the app will be remunerated Be explicit about the implications of data security and privacy issues for the PCP	Explain how app can improve documentation of care Must also be flexible enough to accommodate different patient goals and care plans	Minimize workflow disruption and avoid unnecessary increase in workload through automation and integration with existing technology

^a^PCP: primary care physician.

### Limitations

There are several limitations to this study. First, PCPs were drawn from clinics that were already voluntarily engaged with a regional public health body that aimed to improve the efficiency and effectiveness of primary care. These clinics were therefore likely to be more receptive to public health initiatives (including the app, which would be delivered through a collaborative body that includes the local health district). As such, the PCPs in this study may be more positive than other PCPs in the community. This is particularly important regarding sensitive issues such as remuneration and may explain why this was raised by so few PCPs.

The app was intended to first roll out in Western Sydney, a suburban region with a highly culturally diverse population and areas of socioeconomic disadvantage. As such, recruitment focused on PCPs in that area and results are more likely to reflect the perspectives of PCPs working in that kind of setting. In addition, although the research was carried out by an associate of the team that would eventually develop the app, efforts were made to ensure that PCPs understood the interviewer’s independence and that they did not feel pressure to provide positive responses about apps.

Second, PCPs discussed their attitudes toward hypothetical app features rather than an actual app. As such, these findings reflect more abstract preconceptions and assumptions about apps. This is useful for anticipating potential barriers and engaging PCPs. However, this approach may have also overemphasized PCP openness to changes in workload as it is more likely to reflect aspirational goals of care.

### Conclusions

Diabetes self-management apps that are linked to the patient’s PCP have the potential to be highly effective. However, in reality these interventions are often limited by poor health care provider uptake. This study investigated PCP perspectives on a diabetes app that was integrated with primary care services. PCPs perceived this as more than a technological change; they were concerned about changes in workload, their role in self-management, and the nature of consultations. This research highlighted potential facilitators and barriers to engaging PCPs in the implementation process.

## Appendix

Multimedia Appendix 1 Interview schedule, example summary report presented to primary care physicians during the interview, and Consolidated Criteria for Reporting Qualitative Research checklist.

## Abbreviations

LOTE: language other than English
PCP: primary care physician
