# Supplementary material for: Factors for Supporting Primary Care Physician Engagement With Patient Apps for Type 2 Diabetes Self-Management That Link to Primary Care: Interview Study
Source: JMIR Mhealth Uhealth. 2019 Jan 16;7(1):e11885. doi: 10.2196/11885 (PMC6352005; doi:10.2196/11885)
Supplement: Multimedia Appendix 1 [file mhealth_v7i1e11885_app1.pdf]

Appendix A: Interview schedule

Part 1. Information resources and mobile phone apps

1. What kinds of resources or aids do you use with your patients with diabetes? *[prompt for: pamphlets, websites, phone services.]*

2. What do you think about phone apps for diabetes?

3. We are in the process of designing a smartphone app for to help patients with self-management of diabetes. Each patient's account will be linked to their PCP to facilitate feedback, communication and/or monitoring. There are potentially many benefits to integrating health care providers into the app. However, it is also important that this doesn't place too much burden on the health care providers. How do you think we could balance these two needs?

4. Do you think apps could be useful for your patients who are culturally and linguistically diverse? [*prompt for: Why/why not]*

5. One of the aims of the app is to save the practitioner time and effort where possible whilst maintaining a high quality of care. I will go through some of the possible strategies to achieve this:

    a. Transfer the patient's care plan into the app. For example, blood glucose levels can be assessed automatically by the app using targets set by the doctor.

       Would this be a useful feature? What problems/risks do you forsee with this approach?

    b. Data export function for blood glucose monitoring data/physical activity to be used during PCP consultations.

Would this be a useful feature? How would you use this information in a consultation?

c. Prompt for patient to see their doctor (for example, if they had several very high blood glucose levels) and periodic reminders for check-ups with PCP and eye and foot checks with specialists. *[can include manually implemented and automatic prompts]*

How useful would this feature be? What problems/risks do you think there might be?

6. GoShare Healthcare is an interface that allows PCPs to 'bundle' videos and educational material from a database of materials, and then either email or text these resources to patients. Each patient can receive their own specific bundle for their needs. It has been used in Victoria by a PCPs as a way to improve communication with patients. How would you feel about using this kind of resource to communicate with your patients?

7. I'd like to go back to the patient scenarios we discussed before. I will show you examples of app content or output from three different patients. This information will be similar to what you would receive as a 'summary report' of the patients' self-management and blood glucose readings.

[provide participant with example output for a patient with several low blood glucose readings, one with mostly on-target blood glucose readings, and one with several high blood-glucose readings].

How would this app output change the way you manage these patients? *[prompt for: any key information missing?]*

What kinds of things would you want to feed back into the app?

Part 2: Any additional feedback?

8. Do you have any additional feedback regarding patient self-management or diabetes or how this could be integrated into an app?

Appendix B: Example summary report presented to PCPs during the interview

Summary of blood glucose self-monitoring for [First name] [Last name], DD/MM/YYYY-DD/MM/YYYY

Blood glucose level key

Blood glucose level	Range (mmol/L)	
	Preprandial	Postprandial
Very low	≤2.9	≤2.9
Low	3.0-4.9	3.0-4.9
On-target	5.0-9.9	5.0-11.9
High	10.0-11.9	12.0-13.9
Very high	≥12.0	≥14.0

Proportion of blood glucose readings in each level

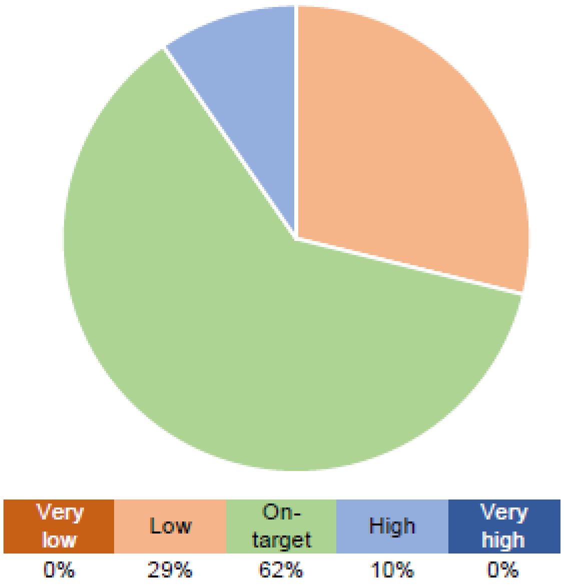

Daily output

Day	Blood glucose level (mmol/L)		
	Fasting	Pre-meal	2 hours post-meal
1	4.1	7.2	7.8
2	4.8		
3		3.4	
4		4.7	
5		9.3	
6		10.2	
7	7.6		9.5
8		6.6	
9	4.2	6.6	12.
10	7.3	5.1	8.0
11			10.7
12		9.9	
13	9.8	5.0	10.0
14	10.	9.6	9.1
15		10.0	
16	8.5		8.5
17		4.0	8.5
18	6.7	4.2	4.1
19	4.0		
20	9.6	10.3	
21		4.4	4.0
22			
23	6.8	8.9	3.3
24	9.6	9.3	
25	3.3		9.8
26	5.6	9.9	8.9
27	9.2	3.0	
28	7.4	2.4	

*NB: Daily results indicate the average of readings in each of the pre/post prandial/fasting categories.

Proportion of blood glucose levels by time of testing

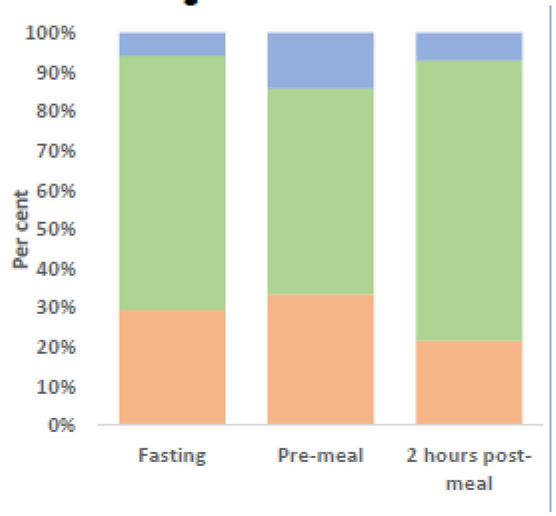

Appendix B: COREQ checklist

Domains 1 and 2 from the Consolidated Criteria for Reporting Qualitative Studies (COREQ)

Developed from:
Tong A, Sainsbury P, Craig J. Consolidated criteria for reporting qualitative research (COREQ): a 32-item checklist for interviews and focus groups. *International Journal for Quality in Health Care*. 2007. Volume 19, Number 6: pp. 349 – 357

**No. Item**	**Guide questions/description**	**Notes**
Personal Characteristics<br><br>***Domain 1: Research team and reflexivity***		
1. Inter viewer/facilitator	Which author/s conducted the interview or focus group?	Interviews were facilitated by the first author (JA).
2. Credentials What were the researcher's credentials? E.g. PhD, MD	JA: B.LibSt (Hons in Psychology)	
3. Occupation	What was their occupation at the time of the study?	At the time of the interviews, JA was a PhD candidate.
4. Gender	Was the researcher male or female?	The researcher was female.
5. Experience and training	What experience or training did the researcher have?	JA was trained in qualitative methods.
Relationship with participants		
6. Relationship established		
7. Participant knowledge of the interviewer Was a relationship established prior to study commencement? JA did not have any direct contact with participants prior the interviews. JA was	What did the participants know about the researcher? e.g. personal goals, reasons for doing the research	Participants were informed that the researcher was interested in interviewing PCPs about diabetes apps as part of her PhD, with a view to informing the design of an app that would be created by Western Sydney Diabetes, a group of collaborating local health authorities.

introduced to participants through the health professionals involved in joint specialist case conferencing.		
8. Interviewer characteristics	What characteristics were reported about the inter viewer/facilitator? e.g. Bias, assumptions, reasons and interests in the research topic	Participants were informed that the researcher was interested in interviewing PCPs about diabetes apps as part of her PhD, with a view to informing the design of an app that would be created by Western Sydney Diabetes, a group of collaborating local health authorities.

**Domain 2: Study design**		
Theoretical framework		
9. Methodological orientation and Theory	What methodological orientation was stated to underpin the study? e.g. grounded theory, discourse analysis, ethnography, phenomenology, content analysis	Methods in this study were based on phenomenology and framework analysis. Phenomenology focuses on individual's experience, and appropriate for our interest in PCPs perceptions and attitudes towards a proposed diabetes app
Participant selection		
10. Sampling	How were participants selected? e.g. purposive, convenience, consecutive, snowball	Participants were purposively sampled to ensure a diverse range of gender, years of experience and cultural backgrounds. Participants were recruited from Western Sydney, an area with culturally and linguistically diverse patient populations. Participants were recruited from a pool of 50 clinics in Western Sydney who had elected to engage in joint specialist case conferencing, an initiative implemented through the Western Sydney Primary Health Network. During case conferencing the PCP discusses diabetes management with the patient, in conjunction with an endocrinologist and a credentialled diabetes educator.

11. Method of approach	How were participants approached? e.g. face-to-face, telephone, mail, email	Participants were approached face-to-face in the PCP's clinic on days that the joint specialist case conferencing team had consultations scheduled in the clinic
12. Sample size	How many participants were in the study?	There were 25 participants in the study.
13. Non-participation	How many people refused to participate or dropped out? Reasons?	There were no participants who dropped out. Some PCPs were approached and indicated interest but ultimately decided not to participate because they had limited time do so.
Setting		
14. Setting of data collection	Where was the data collected? e.g. home, clinic, workplace	Interviews were conducted face-to-face in the PCPs consultation room.
15. Presence of non-participants	Was anyone else present besides the participants and researchers?	Only the participant and researcher were present at the time of the interview.
16. Description of sample	What are the important characteristics of the sample? e.g. demographic data, date	Table 1. Interviews were conducted between November 2017 and June 2018
Data collection		
17. Interview guide	Were questions, prompts, guides provided by the authors? Was it pilot tested?	Interviews were semi-structured and roughly followed the interview schedule shown in Multimedia Appendix 1. Questions asked about how the PCP currently helps patients to self-manage diabetes and their attitudes towards diabetes apps. Participants were also asked for feedback on several specific app features, including:<br>1. A feature that transfers a patient's individualized care plan into the app<br>2. A feature that exports self-monitoring data to PCP software<br>3. Prompts to see their PCP (for example, if there is a pattern of high blood glucose readings)<br>4. Reminders to book cycle of care appointments (for example, PCP check-ups, and eye and foot checks)<br>5. Bundles of educational material including videos that can be sent to the patient<br>6. A 'summary' report of blood glucose self-monitoring to be used by the PCP

		during the consultation (see Appendix 1).
18. Repeat interviews	Were repeat inter views carried out? If yes, how many?	No repeat interviews were carried out.
19. Audio/visual recording	Did the research use audio or visual recording to collect the data?	All interviews except one were audio-recorded and transcribed verbatim by an independent transcription service. One interview was not audio-recorded as the PCP had requested that only notes be taken (although these were transcribed verbatim as much as possible).
20. Field notes	Were field notes made during and/or after the interview or focus group?	Field notes were only made during one interview (see above), for which the participant had requested no audio-recording be made.
21. Duration<br>What was the duration of the inter views or focus group?	Interviews lasted an average 25 minutes.	
22. Data saturation	Was data saturation discussed?	Analysis took place concurrently and iteratively as interviews were conducted. This meant that saturation was discussed with the research team throughout the analysis process. The project team concluded that theoretical saturation was reached after 25 interviews, where variation in PCP attitudes could be adequately explained through three main overarching themes
23. Transcripts returned	Were transcripts returned to participants for comment and/or correction?	Transcripts were not returned to participants for comment and/or correction.
